# Association Between Time From Percutaneous Coronary Intervention to Cancer Surgery and Cardiovascular and Oncological Outcomes

**DOI:** 10.1161/JAHA.124.038569

**Published:** 2025-04-16

**Authors:** Ki Hong Choi, Junghee Lee, Jin Lee, Juhee Cho, Danbee Kang, Hong Kwan Kim

**Affiliations:** ^1^ Division of Cardiology, Department of Internal Medicine, Heart Vascular Stroke Institute, Samsung Medical Center Sungkyunkwan University School of Medicine Seoul Republic of Korea; ^2^ Department of Thoracic and Cardiovascular Surgery, Samsung Medical Center Sungkyunkwan University School of Medicine Seoul Republic of Korea; ^3^ Department of Clinical Research Design and Evaluation, SAIHST Sungkyunkwan University Seoul Republic of Korea; ^4^ Center for Clinical Epidemiology, Samsung Medical Center Sungkyunkwan University School of Medicine Seoul Republic of Korea; ^5^ Trend Sensing & Risk Modeling Center, Institution of Quality of Life in Cancer Samsung Medical Center Seoul South Korea

**Keywords:** cancer, percutaneous coronary intervention, surgery, time, Percutaneous Coronary Intervention, Epidemiology

## Abstract

**Background:**

Patients who undergo noncardiac surgery shortly after percutaneous coronary intervention (PCI) experience higher rates of perioperative ischemic events, but delaying surgery may affect disease staging and influence cancer recurrence. We aimed to evaluate the association between time from PCI to cancer surgery and cardiovascular and oncologic outcomes in patients with early‐stage cancer.

**Methods and Results:**

We included patients with early‐stage cancer with a history of PCI who underwent cancer surgery (N=3621). The patients were divided into 2 groups based on the time between the dates of PCI and cancer surgery (<12 and ≥12 months). We also grouped patients who underwent early surgery and late surgery, defined as patients who underwent surgery ≥1 and <1 month after cancer diagnosis. Outcomes included bleeding, spontaneous myocardial infarction, repeat revascularization, cancer recurrence, and death. The time from PCI to cancer surgery <12 months group had higher bleeding (hazard ratio [HR], 1.30 [95% CI, 1.18–1.32]), spontaneous myocardial infarction (HR,1.96 [95% CI, 1.32–2.92]), cancer recurrence (HR, 1.26 [95% CI, 1.01–1.56]), and mortality (HR, 1.23 [95% CI, 1.04–1.44]) compared with the ≥12 months group. Among the time from PCI to cancer surgery <12 months group, the early‐surgery group had lower cancer recurrence risk than those who underwent late surgery (HR, 0.70 [95% CI, 0.49–0.99]) without differences in bleeding and cardiac outcome.

**Conclusions:**

Although patients who undergo surgery within 12 months of PCI have higher risks of bleeding and cardiovascular events, delaying surgery may increase the risk of cancer recurrence. Therefore, the timing of surgery should be a personalized decision, weighing the risks of cardiovascular complications against the potential oncologic outcomes.

**Registration:**

URL: https://www.clinicaltrials.gov; Unique Identifier: NCT06357000.

**Nonstandard Abbreviations and Acronyms**
DES: drug‐eluting stent
IPTW: inverse probability of treatment weighting

**Clinical Perspective**

**What Is New?**

- Patients who undergo surgery within 12 months of percutaneous coronary intervention have higher risks of bleeding and cardiovascular events.
- However, delaying surgery may increase the risk of cancer recurrence.

**What Are the Clinical Implications?**

- The timing of surgery should be a personalized decision, weighing the risks of cardiovascular complications against the potential oncologic outcomes.

Coronary artery disease (CAD) and cancer remain the 2 leading causes of death worldwide.^1^ The association between CAD and cancer is attributable to shared risk factors, including advanced age, smoking, and atherosclerotic risk score.^2^, ^3^ Recently, advancements in patient care, particularly the improvement of medical treatment and revascularization procedures for cardiovascular diseases, have significantly reduced mortality rates in patients with CAD.^4^, ^5^, ^6^ As a result, the population of patients with CAD who are diagnosed with cancer after percutaneous coronary intervention (PCI) has increased. Considering that cancer itself and its treatment can affect atherosclerotic cardiovascular disease aggravation, and that cardiovascular disease is the primary cause of mortality among cancer survivors, perioperative management in patients with early‐stage cancer with preexisting CAD undergoing prior PCI is vital.^7^, ^8^, ^9^

Several studies have demonstrated that patients who undergo noncardiac surgery shortly after PCI experience higher rates of perioperative ischemic events.^10^, ^11^, ^12^, ^13^ In this regard, the current guidelines recommend optimally delaying elective noncardiac surgery for 6 months after drug‐eluting stent (DES) implantation.^14^, ^15^ Additionally, lifelong antiplatelet therapy is mandatory for preventing future ischemic events after PCI. Therefore, bleeding risks should be considered when performing noncardiac surgery in patients with concomitant CAD and cancer.^16^ On the other hand, surgical delay due to recent PCI history may lead to upstaging of cancer stage and affect recurrence and mortality after cancer diagnosis.^17^ However, there are limited data comparing bleeding, cardiovascular, and oncologic outcomes according to the PCI and surgical timing. Therefore, the current study aimed to evaluate the association between time from PCI to cancer surgery and cardiovascular and oncologic outcomes in patients with early‐stage cancer.

## METHODS

### Data Sharing Statement

The participants of this study did not give written consent for their data to be shared publicly, so due to the sensitive nature of the research, supporting data are not available.

### Study Population and Design

**Figure 1:**
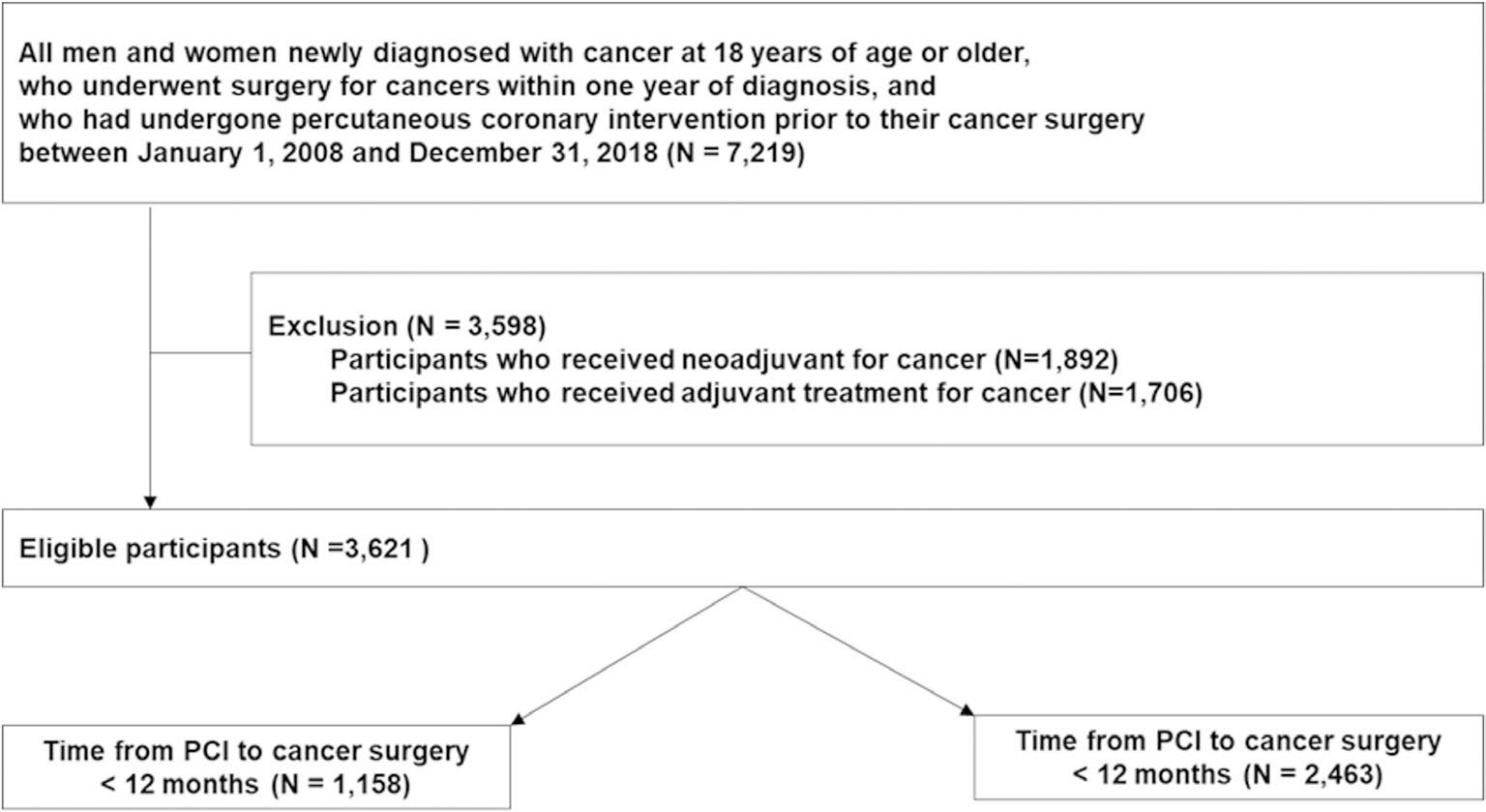
Flowchart. PCI indicates percutaneous coronary intervention.

We conducted a retrospective, population‐based cohort study using data from the Korean National Health Insurance Service database. The Korean National Health Insurance Service is a single insurer in Korea, covering approximately 97% of the population, and 3% of the beneficiaries are covered by the Medical Aid Program. To evaluate the association between the time from PCI to surgery and clinical outcomes, we included men and women aged ≥18 years with history of PCI (procedure codes: M6551–M6572, O1640–O1649, OA640–OA649) who were newly diagnosed with cancer after PCI and underwent cancer surgery within 1 year following cancer diagnosis, between January 1, 2008 and December 31, 2018 (N=7219). To select the patients with early‐stage cancer, we excluded participants who received neoadjuvant (N=1892) or adjuvant treatment (N=1706) for cancer. Finally, 3621 participants were included (Figure 1). The institutional review board of the Samsung Medical Center approved this study (SMC‐201912064) and waived the requirement for informed consent due to its retrospective nature. In accordance with the Declaration of Helsinki, the research protocol was registered in clinicaltrials.gov (NCT06357000).

### Measurement

The Korean National Health Insurance Service database contains claims data for inpatient and outpatient visits, procedures, and prescriptions coded using the *International Classification of Diseases*, *Tenth Revision* (*ICD‐10*) and the *Korean Drug and Anatomical Therapeutic Chemical Codes*.^18^ The Korean National Health Insurance Service routinely audits the claims, and the data are considered reliable and have been used in numerous peer‐reviewed publications.

Cancer is defined by a cancer‐specific insurance claim code (code V193) with a C code that corresponds to the *ICD‐10* code for cancer. Cancer surgery was categorized according to a specific procedure code. We calculated the number of months between the dates of PCI and cancer surgery and divided the patients into 2 groups based on the time from PCI to cancer surgery: <12 and ≥12 months.

The date of cancer diagnosis was defined as the date on which the recipient became eligible for special insurance benefits. We then calculated the difference between the dates of cancer diagnosis and surgery. Among the patients who underwent PCI within 12 months before cancer surgery, we grouped patients who underwent early surgery, defined as patients who underwent surgery within 1 month after cancer diagnosis, and late surgery, defined as patients who underwent surgery 1 month after cancer diagnosis.

Bleeding events, adverse cardiovascular events, cancer recurrences, and death were considered as the clinical outcomes. Bleeding requiring transfusion was defined by the transfusion codes (X2021, X2022, X2031, X2032, X2091, X2092, X2111, X2112, X2131, X2132). Spontaneous myocardial infarction (MI) was defined as hospitalization for MI (*ICD‐10* codes I21 and I22) as the primary or secondary cause, and repeat revascularization was defined as the presence of a procedure code after cancer surgery. Cancer recurrence was defined as the presence of either chemotherapy or radiation therapy that began >6 months after surgery completion.^19^ Vital status was obtained from death certification collected by Statistics Korea at the Ministry of Strategy and Finance of South Korea.^20^

For the covariate, we included age, sex, comorbidities, income, and residential area at baseline. Comorbidities during the year before cancer surgery was obtained from claims data defined using *ICD‐10* codes. We included diabetes, hypertension, dyslipidemia, and heart failure, which were defined as the presence of at least 1 code during the year before baseline (with medication), as comorbidities. Because smoking status was crucial for both cancer and cardiac outcomes, we used the national health screening examinations data, which is a standardized program provided to all insured people every 2 years.^21^ In our study, 76% of participants had national health screening examinations data within 4 years before cancer diagnosis, and we obtained their smoking status using a standard questionnaire from the data set.

We also identified antiplatelet medications, including aspirin and P2Y12 inhibitors (clopidogrel, ticagrelor, and prasugrel), from prescription claims data using the *Korean Drug and Anatomical Therapeutic Chemical Codes*. Clinical characteristics, type of cancer, and type of surgery were defined using *ICD‐10* and procedure codes. We also classified the surgical risk of bleeding according to the European Society of Cardiology/European Society of Anesthesiology based on the type of cancer and surgery.

### Statistical Analysis

Continuous variables are presented as means and standard deviations or medians and interquartile ranges and were compared using the *t* test. Categorical variables are presented as numbers and proportions and were compared using the χ^2^ test. For the incidence of bleeding requiring transfusion, spontaneous MI, revascularization, cancer recurrence, and all‐cause mortality, the cumulative events from surgery to the last follow‐up were estimated using the Kaplan‐Meier method. We used Cox proportional hazards regression models to compare the hazard ratios (HRs) with 95% CIs of the PCI <12 and ≥12 months groups. All participants were followed up from the time of cancer surgery until either an incident event, death, or 5 years postsurgery. The proportionality of the hazards was confirmed by visual inspection of the log‐minus‐log plots and Schoenfeld residuals.

An inverse probability of treatment weighting (IPTW) model was used to balance baseline characteristics between both groups. The propensity score for receiving surgery within 12 months after PCI used in the IPTW model was estimated using multivariable logistic regression analysis, including age, sex, income percentile, residential area, clinical presentation at the time of PCI, diabetes, hypertension, heart failure, cancer type, surgical risk, and time from cancer diagnosis to surgery. Differences in baseline covariables between the 2 groups were evaluated before and after the IPTW using an absolute standardized mean difference with a value of >0.2 indicating a significant difference.^22^ To further investigate the influence of the time from PCI to surgery, we modeled the time from PCI to surgery as a continuous variable using restricted cubic splines. After checking whether the hypothesis for *P* for nonlinearity was rejected (*P*>0.05), we calculated *P* for linearity, which was calculated using time from PCI to surgery as a continuous variable.

We also compared the risk of bleeding requiring transfusion, spontaneous MI, revascularization, cancer recurrence, and all‐cause mortality according to the surgical timing among patients receiving recent PCI (<12 months) using Cox regression with IPTW methods. The IPTW model included age; sex; income percentile; residential area; clinical presentation at the time of PCI; diabetes; hypertension; heart failure; dyslipidemia; aspirin, clopidogrel, prasugrel, and ticagrelor use; cancer type; and surgical risk. We performed subgroup analysis by surgical risk and type of cancer (colorectal, gastric cancer, lung cancer, thyroid cancer, genitourinary track cancer, and others). Furthermore, we performed a sensitivity analysis on risk of cardiovascular events and cancer outcomes in patients receiving surgery within 6 months after PCI. For bleeding, MI, revascularization, and cancer recurrence, we calculated the sub‐HR using the Fine‐Gray regression model to account for the competing risks of death.^23^

All *P* values were 2‐tailed, and a *P* value <0.05 was considered statistically significant. Analyses were performed using SAS Visual Analytics (SAS Institute) and R 4.1.2 (R Foundation for Statistical Computing, Vienna, Austria).

## RESULTS

### Baseline Characteristics

The mean patient age was 68 years, and 77% of the patients were men. The most frequent cancers were stomach and colorectal cancers. Among the patients with cancer who underwent PCI before cancer surgery, 32% underwent PCI <12 months before cancer surgery (Table 1). Distributions of the time from PCI to cancer surgery in patients from both groups are depicted in Figure S1. Patients with a PCI‐to‐cancer‐surgery interval of <12 months experienced a significantly longer duration from cancer diagnosis to surgery compared with those with an interval of ≥12 months (21.2 versus 15.1 days, respectively; standardized mean difference=−0.309). After IPTW, there was no evidence of inequality in baseline characteristics, comorbidities, or medication history between the 2 groups (all standardized mean differences <0.2).

**Table 1:** Characteristics of the Study Population According to the Time of Prior PCI to Surgery

Characteristic	Time from PCI to cancer surgery		SMD	IPTW SMD
	<12 mo (N=1158)	≥12 mo (N=2463)		
Age, y	67.8 (9.1)	68.3 (9.3)	0.054	0.003
Sex, men	897 (77.5)	1876 (76.2)	−0.031	−0.026
Income percentile				
Medical aid	77 (6.6)	136 (5.5)	−0.047	−0.033
>30th	202 (17.4)	418 (17.0)	−0.013	−0.075
30th–70th	419 (36.2)	928 (37.7)	0.141	0.049
<70th	438 (37.8)	929 (37.7)	−0.002	−0.013
Unknown	22 (1.9)	52 (2.1)	0.015	0.042
Residential area, metropolitan	721 (62.3)	1569 (63.7)	0.030	0.043
Ever smoker (N=2651)	194 (25.6)	352 (18.6)	−0.169	−0.077
Comorbidities				
Charlson Comorbidities Index	6.60 (2.70)	6.06 (2.87)	−0.194	−0.107
Diabetes	751 (64.9)	1337 (54.3)	−0.217	−0.061
Hypertension	245 (21.2)	476 (19.3)	−0.046	−0.026
Dyslipidemia	1051 (90.8)	2257 (91.6)	0.031	0.057
Heart failure	80 (6.9)	136 (5.5)	−0.057	−0.006
Clinical presentation at the time of PCI				
MI	281 (24.3)	749 (30.4)	0.138	0.004
Angina	877 (75.7)	1714 (69.6)	−0.138	−0.004
Antiplatelet therapy	1062 (91.7)	1080 (43.8)	−1.192	−0.004
Aspirin	526 (45.4)	641 (26.0)	−0.413	−0.001
Clopidogrel	520 (44.9)	455 (18.5)	−0.593	−0.004
Prasugrel	6 (0.5)	3 (0.1)	−0.070	−0.003
Ticagrelor	33 (2.8)	4 (0.2)	−0.222	−0.004
Cancer type				
Stomach	463 (40.0)	847 (34.4)	−0.116	−0.046
Colorectal	283 (24.4)	560 (22.7)	−0.040	−0.066
Thyroid	144 (12.4)	346 (14.0)	0.048	0.007
Genitourinary tract	170 (14.7)	409 (16.6)	0.053	0.005
Lung	47 (4.1)	128 (5.2)	0.054	0.109
Others	51 (4.4)	173 (7.0)	0.113	0.121
ESC/ESA surgical risk				
Low	158 (13.6)	422 (17.1)	0.098	0.052
Moderate–high	1000 (86.4)	2041 (82.9)	−0.098	−0.052
Time from PCI to cancer surgery, mo			2.226	2.070
Median (IQR)	3.3 (1.4–7.4)	38.2 (23.3–60.5)		
Mean±SD	4.5±3.6	44.1±24.9		
Time from PCI to cancer diagnosis, mo			2.216	2.075
Median (IQR)	2.4 (1.1–6.6)	37.2 (22.6–59.8)		
Mean±SD	3.9±3.5	43.3±24.9		
Time from cancer diagnosis to surgery, d			−0.309	−0.182
Median (IQR)	21.2 (0–54.5)	15.1 (0–30.3)		
Mean±SD	39.3±54.5	24.2±42.4		

Values are presented as n (percent), median (IQR), or mean±SD. ESC/ESA indicates European Society of Cardiology/European Society of Anesthesiology; IPTW, inverse probability of treatment weighting; IQR, interquartile range; MI, myocardial infarction; PCI, percutaneous coronary intervention; and SMD, standardized mean difference.

### Risk of Adverse Events in Patients Who Underwent PCI Within 12 Months Before Surgery

#### Bleeding Events

**Figure 2:**
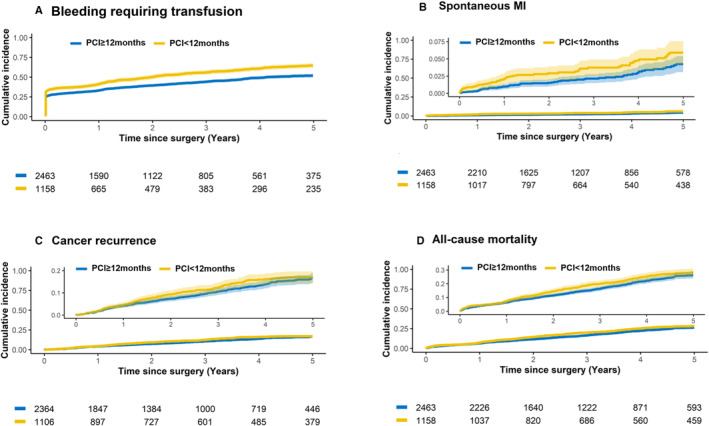
Kaplan–Meier curves for clinical outcomes. Kaplan–Meier curves showing the incidence of bleeding requiring transfusion (**A**), spontaneous myocardial infarction (**B**), cancer recurrence (**C**), and all‐cause mortality (**D**). MI indicates myocardial infarction; and PCI, percutaneous coronary intervention.

In‐hospital bleeding requiring transfusion was 17.6% and 13.7% in the time from PCI to cancer surgery <12 and ≥12 months groups, respectively (*P*=0.002; Figure S2). The time from PCI to cancer surgery ≥12 months group had a bleeding event rate of 21.1 per 100 person‐years, whereas the <12 months group had a rate of 29.2 per 100 person‐years (Figure 2A). The IPTW HR for bleeding events in the time from PCI to cancer surgery <12 months group compared with the ≥12 months group was 1.30 (95% CI, 1.18–1.32; Table 2).

**Table 2:** Cardiovascular, Bleeding, and Cancer Outcomes According to the Time of Prior PCI

Outcome	Time from PCI to cancer surgery <12 mo (N=1158)	Time from PCI to cancer surgery ≥12 mo (N=2463)
Bleeding requiring transfusion		
No. of cases (IR per 100 py)	681 (29.2)	1097 (21.1)
Crude HR (95% CI)	1.42 (1.29–1.56)	Reference
IPTW* HR (95% CI)	1.30 (1.18–1.43)	Reference
Competing risk sub‐HR† (95% CI)	1.36 (1.24–1.48)	Reference
Spontaneous MI		
No. of cases (IR per 100 py)	49 (1.3)	61 (0.8)
Crude HR (95% CI)	1.76 (1.21–2.56)	Reference
IPTW* HR (95% CI)	1.96 (1.32–2.92)	Reference
Competing risk sub‐HR† (95% CI)	1.55 (1.06–2.27)	Reference
Repeat revascularization		
No. of cases (IR per 100 py)	121 (3.3)	180 (2.5)
Crude HR (95% CI)	1.47 (1.17–1.85)	Reference
IPTW* HR (95% CI)	1.38 (1.09–1.76)	Reference
Competing risk sub‐HR† (95% CI)	1.32 (1.05–1.67)	Reference
Cancer recurrence		
No. of cases (IR per 100 py)	140 (3.7)	239 (3.3)
Crude HR (95% CI)	1.28 (1.04–1.58)	Reference
IPTW* HR (95% CI)	1.26 (1.01–1.56)	Reference
Competing risk sub‐HR† (95% CI)	1.11 (0.90–1.37)	Reference
All‐cause mortality		
No. of cases (IR per 100 py)	271 (7.0)	460 (6.2)
Crude HR (95% CI)	1.31 (1.13–1.53)	Reference
IPTW* HR (95% CI)	1.23 (1.04–1.44)	Reference

HR indicates hazard ratio; IPTW, inverse probability of treatment; IR, incidence rate; MI, myocardial infarction; PCI, percutaneous coronary intervention; py, person‐years; and sub‐HR, subdistribution HR.

* IPTW includes age, sex, income percentile, residential area, clinical presentation at the time of PCI, diabetes, hypertension, heart failure, cancer type, surgical risk, and time from cancer diagnosis to surgery.

† Sub‐HRs were modeled with all‐cause mortality as a competing risk.

**Figure 3:**
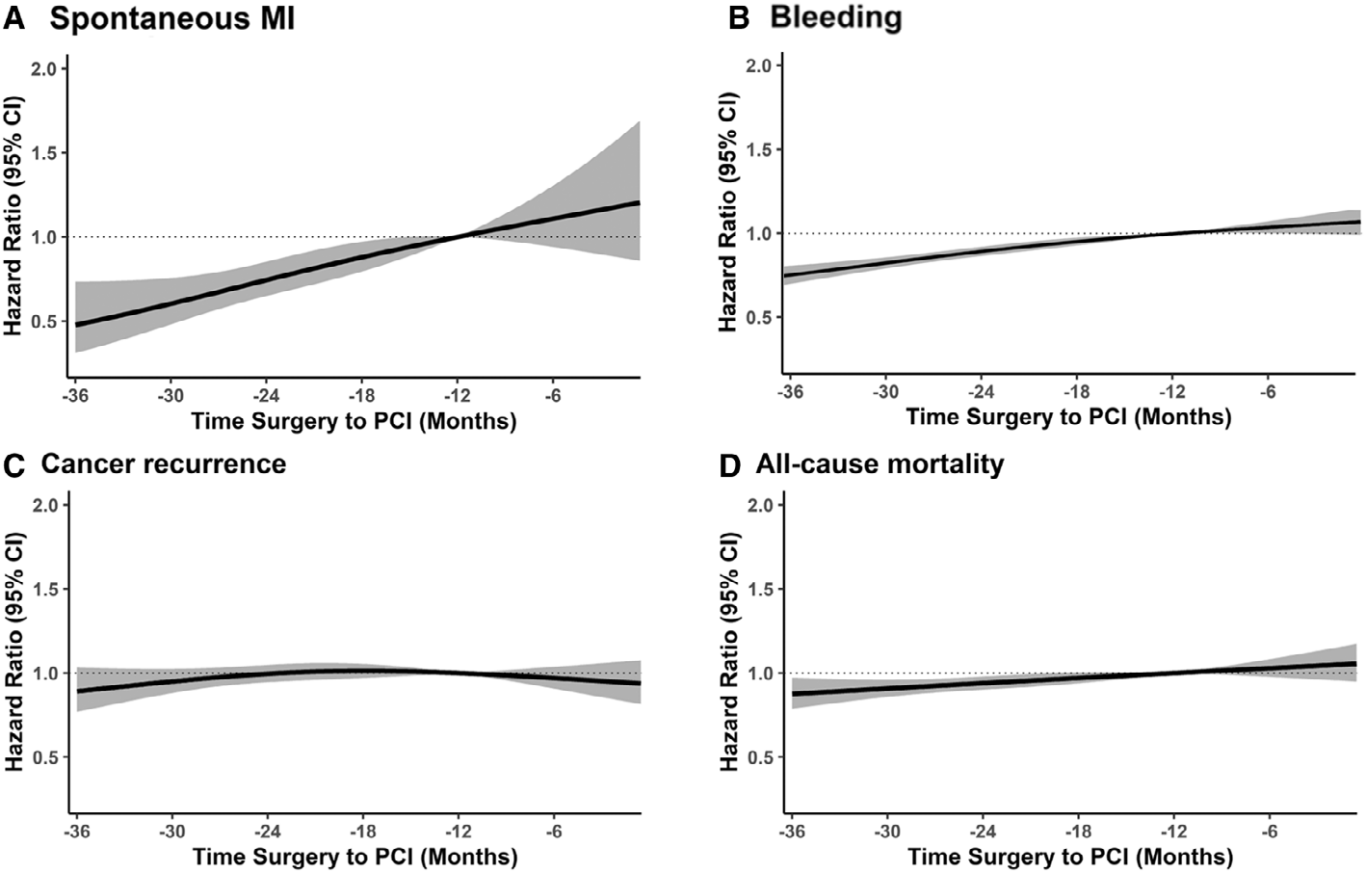
Multivariable‐adjusted hazard ratios for clinical outcomes by PCI‐to‐surgery interval. Multivariable‐adjusted HRs with 95% CIs for bleeding requiring transfusion (**A**), spontaneous myocardial infarction (**B**), cancer recurrence (**C**), and all‐cause mortality (**D**) according to the PCI‐to‐surgery interval. The curves represent the IPTW HR (solid line) and 95% CI (gray area) for each outcome based on restricted cubic splines for the PCI‐to‐surgery interval with knots at the 5th, 35th, 65th, and 95th percentiles of the sample distributions. HR indicates hazard ratio; IPTW, inverse probability of treatment; MI, myocardial infarction; and PCI, percutaneous coronary intervention.

The risk of bleeding increased linearly as the time from PCI to cancer surgery decreased (*P* for linearity <0.01; Figure 3A).

#### Ischemic Events

During the follow‐up (median: 2.8 years, interquartile range: 1.4–4.9 years), the rates of spontaneous MI were 1.3 and 0.8 per 100 person‐years for the time from PCI to cancer surgery <12 months and ≥12 months groups, respectively (Figure 2B). Spontaneous MI risk was significantly higher in the time from PCI to cancer surgery <12 months group than in the ≥12 months group (IPTW HR, 1.96 [95% CI, 1.32–2.92]). Repeat revascularization also showed a trend similar to that of spontaneous MI (Table 2). The incidence of spontaneous MI increased linearly with a shorter time from PCI to cancer surgery (*P* for linearity <0.01; Figure 3B).

#### Cancer Recurrence

The incidence of cancer recurrence was 3.7 and 3.3 cases per 100 person‐years in the time from PCI to cancer surgery <12 and ≥12 months groups, respectively (Figure 2C). The IPTW HR for cancer recurrence in the time from PCI to cancer surgery <12 months group was 1.26 (95% CI, 1.01–1.56) compared with the ≥12 months group (Table 2). In competing risk analysis, the results were also similar (sub‐HR, 0.57 [95% CI, 0.37–0.87]). The risk of cancer recurrence increased linearly as the time from PCI to cancer surgery was shorter until 12 months (*P* for linearity <0.01; Figure 3C).

#### Mortality

In‐hospital mortality rates for the time from PCI to cancer surgery <12 and ≥12 months groups were 2.0% and 1.3%, respectively (*P*=0.12; Figure S2). In regard to all‐cause mortality, the IPTW HR was 1.23 (95% CI, 1.04–1.44) in the time from PCI to cancer surgery <12 months group compared with the ≥12 months group (Table 2 and Figure 2D). The risk of all‐cause mortality was higher, with a shorter time from PCI to cancer surgery (*P* for linearity <0.01; Figure 3D).

#### Subgroup Analysis

There was no significant interaction between the timing of prior PCI and surgical risk as well as type of cancer in terms of the risk of ischemia, bleeding, cancer recurrence, or all‐cause mortality (Tables S1 and S2).

#### Sensitivity Analysis

Sensitivity analyses showed that the HR for cardiovascular events was significantly higher in the group receiving PCI <6 months after cancer surgery compared with those receiving PCI ≥6 months after cancer surgery (Table S3). Additionally, the effect size was larger than that of the 12‐month cutoff.

### Risk of Adverse Events According to Surgical Timing in Patients Who Underwent Recent PCI

The baseline clinical characteristics between the time from cancer diagnosis to surgery <1 month (early surgery) and ≥1 month (late surgery) are described in Table S4. Among the population with the time from PCI to cancer surgery <12 months, the early‐surgery group had similar risk of bleeding events as the late‐surgery group (IPTW HR, 0.97 [95% CI, 0.84–1.14]; Table 3). Moreover, there was no significant difference between the 2 subgroups in spontaneous MI risk and repeat revascularization. In contrast, patients who underwent early surgery had lower cancer recurrence risk than those who underwent late surgery after adjustment for baseline difference including cancer severity (IPTW HR, 0.70 [95% CI, 0.49–0.99]; Table 3).

**Table 3:** Cardiovascular, Bleeding, and Cancer Outcomes According to Surgical Timing Among Patients Receiving Recent PCI (<12 mo)

Outcome	Time of cancer diagnosis to surgery <1 mo (early surgery) (N=681)	Time of cancer diagnosis to surgery ≥1 mo (late surgery) (N=477)
Bleeding requiring transfusion		
No. of cases (IR per 100 py)	400 (29.3)	281 (29.0)
Crude HR (95% CI)	1.02 (0.87–1.19)	Reference
IPTW* HR (95% CI)	0.97 (0.83–1.14)	Reference
Competing risk sub‐HR† (95% CI)	0.97 (0.83–1.14)	Reference
Spontaneous MI		
No. of cases (IR per 100 py)	33 (1.5)	16 (1.0)
Crude HR (95% CI)	1.49 (0.82–2.70)	Reference
IPTW* HR (95% CI)	1.22 (0.66–2.44)	Reference
Competing risk sub‐HR† (95% CI)	1.16 (0.63–2.14)	Reference
Repeat revascularization		
No. of cases (IR per 100 py)	66 (3.1)	55 (3.7)
Crude HR (95% CI)	0.86 (0.60–1.24)	Reference
IPTW† HR (95% CI)	0.82 (0.56–1.18)	Reference
Competing risk sub‐HR† (95% CI)	1.16 (0.79–1.70)	Reference
Cancer recurrence		
No. of cases (IR per 100 py)	71 (3.2)	69 (4.4)
Crude HR (95% CI)	0.75 (0.54–1.04)	Reference
IPTW* HR (95% CI)	0.70 (0.49–0.99)	Reference
Competing risk sub‐HR† (95% CI)	0.57 (0.37–0.87)	Reference
All‐cause mortality		
No. of cases (IR per 100 py)	162 (7.1)	109 (6.8)
Crude HR (95% CI)	1.05 (0.82–1.34)	Reference
IPTW* HR (95% CI)	1.10 (0.85–1.41)	Reference

HR indicates hazard ratio; IPTW, inverse probability of treatment; IR, incidence rate; MI, myocardial infarction; PCI, percutaneous coronary intervention; py, person‐years; and sub‐HR, subdistribution HR.

* IPTW includes age, sex, income percentile, residential area, clinical presentation at the time of PCI, diabetes, hypertension, heart failure, dyslipidemia, aspirin, clopidogrel, prasugrel, ticagrelor, cancer type, and surgical risk.

† Sub‐HRs were modeled with all‐cause mortality as a competing risk.

## DISCUSSION

This large national cohort study evaluated comprehensive outcomes, including cardiovascular and bleeding events, cancer recurrence, and mortality according to the interval between PCI and cancer surgery. We found that patients with early‐stage cancer with a shorter duration between preoperative PCI and cancer surgery had a longer duration from cancer diagnosis to surgery and significantly higher risks of bleeding, cardiovascular events, cancer recurrence, and all‐cause mortality. These trends were consistent regardless of the surgical risk. Among patients who recently underwent PCI (<12 months) before surgery, those who underwent early surgery (time from cancer diagnosis to surgery <1 month) had lower cancer recurrence risk, without a significant increase in cardiovascular or bleeding events, than those who underwent late surgery.

Cancer is associated with increased ischemic event risks via activation of the coagulation system by tumor cells.^24^ Additionally, patients with cancer generally tend to have increased risks of bleeding, which can be related to local tumor invasion, tumor angiogenesis, systemic effects of the cancer itself, or oncology therapies.^25^ According to the Academic Research Consortium for High Bleeding Risk criteria, active malignancy (defined as a diagnosis within the previous 12 months or ongoing active cancer therapy, including surgery, chemotherapy, or radiotherapy) is considered 1 of the major risks. In a study of patients who underwent PCI in the US Nationwide Readmission Database (N=1 933 324), those with active cancer at the time of PCI (N=18 090) had an increased risk of readmission for acute MI or bleeding than those without cancer following PCI.^26^ Additionally, in a PCI registry, patients with a history of cancer had an increased risk of cardiac mortality and bleeding events.^27^ However, both studies focused on outcomes according to cancer history at the time of PCI and not on PCI history at the time of cancer surgery. Although a previous study identified that patients who underwent PCI before surgery for lung cancer were at higher risk of ischemic events, the study focused only on ischemic events and was limited to patients with lung cancer.^28^ Contrarily, the current study had the major strength of comprehensively analyzing both cardiovascular and oncologic outcomes, such as spontaneous MI, repeat revascularization, and cancer recurrence. Moreover, our study included patients with various types of cancers, suggesting broader clinical implications.

Consistent with the findings of previous studies, we found that patients with a shorter interval between preoperative PCI and cancer surgery had a significantly higher risk of ischemic events. Furthermore, we found that perioperative in‐hospital bleeding risk was higher in patients with time from PCI to cancer surgery <12 months than in those with ≥12 months. Based on our findings, cancer diagnosed within 1 year after PCI should be acknowledged as an independent risk factor for ischemia and bleeding when deciding to undergo surgery. However, bleeding from intraoperative procedures can be controlled with modern surgical techniques and advancements in surgical devices.^29^ In the current study, there was no increase in the in‐hospital mortality rate, despite increased bleeding requiring transfusion. Given the increasing number of patients with both CAD and cancer, further studies are warranted to test the added value of early‐stage cancer in predicting bleeding or ischemic events in patients undergoing PCI.

Cancer progression and outcomes are influenced by various factors including tumor type, stage, biology, patient‐specific characteristics, and treatment modalities. In this study, patients who had PCI within 12 months of surgery had longer delays before surgery than those who had PCI at least 12 months later. We also found that shorter times between PCI and surgery might be associated with increased cancer recurrence. This result suggests that delayed cancer surgery due to a recent PCI history might be associated with an increased risk of oncologic recurrence in patients with early‐stage cancer requiring surgery. It is also possible that surgeons might have restricted the extent of resection for this localized stage of cancer because of concerns about the risk of ischemia or bleeding in this population, resulting in worse oncologic outcomes. Further research with a larger and more homogeneous patient population, longer follow‐up periods, and detailed cancer‐specific analyses is warranted to gain a deeper understanding of this complex relationship.

Surgical oncologists often encounter patients diagnosed with early‐stage cancer shortly after PCI with a DES. The current guidelines recommend delaying nonemergent surgery for 6 months after DES implantation because of the increased ischemic risk of stopping dual‐antiplatelet therapy.^14^, ^30^ However, delaying cancer surgery may increase the stage of cancer or make it inoperable if the cancer type is aggressive.^31^ Moreover, with the recent advances in DES technology and cumulative evidence for the safety of shorter‐duration dual‐antiplatelet therapy in the current‐generation DES era, it might be possible to operate on cancer after stopping dual‐antiplatelet therapy even in a short period of time from PCI.^32^, ^33^ Nevertheless, there have been limited data on the prognostic impact of surgical timing on cardiovascular and oncologic outcomes in patients with a recent history of PCI. Interestingly, among patients with a recent history of PCI (within 12 months), we found that those undergoing early cancer surgery (cancer diagnosis to surgery time <1 month) had a lower risk of cancer recurrence than those undergoing late cancer surgery, without an increase in cardiovascular or bleeding events. Our findings were consistent with the results of a previous meta‐analysis demonstrating that a 1‐month delay in cancer treatment was associated with an increase in mortality across all common forms of cancer.^17^ Therefore, surgical oncologists could consider not delaying cancer surgery to reduce cancer recurrence in patients with early‐stage cancer who have undergone PCI within 1 year before cancer diagnosis, although ischemic and bleeding risks in those patients are high.

### Study Limitations

Our study has several limitations. First, as a retrospective analysis of administrative data, our study lacked detailed clinical information such as cancer stage and procedural complexity of PCI. However, we did our best to enroll a group of patients with similar cancer stages by excluding those who received neoadjuvant or adjuvant treatment. Second, we did not determine whether the patients received a bare‐metal or DES, which may have affected the results. Third, we did not have data on whether or when patients stopped antiplatelet therapy during the perioperative period. Although we acknowledge that perioperative management of antiplatelet therapy is an important factor influencing surgical outcomes, our study's primary focus is on the timing of surgery after PCI. The use of antiplatelet agents may act as a mediator in this relationship, potentially contributing to the increased risks seen with earlier surgeries. Further studies are needed to explore the specific role of antiplatelet discontinuation in this context. Fourth, cancer recurrence was defined as patients requiring systemic treatments and/or radiation 6 months after surgery to reduce false positives, but this definition may potentially underestimate early recurrences. Future studies may consider capturing the full spectrum of early recurrence while maintaining accuracy. Fifth, due to the limitations of the claims data set, we could not identify whether PCI itself may have contributed to the cancer diagnosis. In particular, the current data set could not distinguish patients who were diagnosed with cancer during the workup for coronary artery disease or coronary artery disease discovered during the diagnostic process of cancer. However, in the current study, there are no cases of cancer before PCI, and longer median and mean times from PCI to cancer surgery compared with the cancer diagnosis to surgery times suggest that most patients received their PCI before their cancer diagnosis. The sixth limitation of this study is that it was performed in Korean, which may restrict the generalizability of the findings to other populations. To further validate the findings, future studies in more diverse populations are necessary.

## CONCLUSIONS

In patients with early‐stage cancer with preexisting CAD who underwent prior PCI, a shorter duration before cancer surgery significantly increased the risk of bleeding, cardiovascular events, oncological recurrence, and all‐cause mortality. Among patients who underwent PCI within 12 months before surgery, those who underwent surgery within 1 month had lower cancer recurrence risk without an increase in cardiovascular or bleeding events than those who underwent surgery after 1 month. The findings offer significant clinical insights, underscoring the necessity for a balanced approach to oncologic and cardiovascular risks when determining the optimal timing of cancer surgery after PCI. Further research is required to enhance perioperative management strategies and improve outcomes for this high‐risk population.

## Sources of Funding

This research was supported by a grant of Patient‐Centered Clinical Research Coordinating Center funded by the Ministry of Health and Welfare, Republic of Korea (grant number: HC23C0170).

## Disclosures

None.

## Supporting information

Tables S1–S4
Figures S1–S2
