# Supplementary material for: Association Between Time From Percutaneous Coronary Intervention to Cancer Surgery and Cardiovascular and Oncological Outcomes
Source: J Am Heart Assoc. 2025 Apr 16;14(9):e038569. doi: 10.1161/JAHA.124.038569 (PMC12184590; doi:10.1161/JAHA.124.038569)
Supplement: Supplementary file 1 — Tables S1–S4 Figures S1–S2 [file JAH3-14-e038569-s001.pdf]

Supplemental Material

Table S1. Hazard ratio of PCI < 12 months for cardiovascular, bleeding, and cancer outcomes compared to PCI ≥12 months according to time of previous PCI by surgical risk

	Low surgical risk* IPTW† HR (95% CI)	Moderate-high surgical risk* IPTW† HR (95% CI)	***P for interaction***
**Bleeding requiring transfusion**	1.03 (0.68-1.55)	1.27 (1.14-1.41)	0.98
**Spontaneous MI**	1.32 (0.42-4.12)	2.18 (1.39-3.40)	0.23
**Repeat revascularization**	1.72 (1.01-2.94)	1.36 (1.04-1.79)	0.31
**Cancer recurrence**	1.57 (0.72-3.40)	1.17 (0.93-1.48)	0.76
**All-cause mortality**	1.21 (0.47-3.11)	1.18 (1.00-1.29)	0.70

CI, confidence interval; ESC/ESA, European Society of Cardiology/European Society of Anaesthesiology; HR, Hazard ratio; MI, Myocardial infarction; PCI, percutaneous coronary intervention; IPTW, Inverse probability of treatment

*Surgical risk was defined ESC/ESA surgical risk.

†IPTW included age, sex, income percentile, residential area, clinical presentation at the time of PCI, diabetes, hypertension, heart failure, cancer type, surgical risk and time from cancer diagnosis to surgery.

Table S2. Hazard ratio of PCI < 12 months for cardiovascular, bleeding, and cancer outcomes compared to PCI ≥12 months according to time of previous PCI by subgroup

	IPTW* HR (95% CI)
**Bleeding requiring transfusion**	
Colorectal	1.45 (1.19-1.76)
Gastric	1.36 (1.14-1.63)
Lung	1.02 (0.79-1.31)
Thyroid	0.91 (0.58-1.43)
Genitourinary tract	1.68 (0.91-3.09)
Others	1.39 (0.87-2.21)
P for interaction	0.50
**Spontaneous MI**	
Colorectal	1.58 (0.66-3.79)
Gastric	3.42 (1.68-6.96)
Lung	2.49 (1.00-6.18)
Thyroid	1.78 (0.48-6.56)
Genitourinary tract	4.80 (0.40-57.72)
Others	1.51 (0.29-7.79)
P for interaction	0.62
**Repeat revascularization**	
Colorectal	1.12 (0.66-1.90)
Gastric	1.74 (1.14-2.67)
Lung	1.62 (0.87-3.02)
Thyroid	1.77 (0.99-3.16)
Genitourinary tract	0.88 (0.27-2.86)
Others	2.56 (0.91-7.19)
P for interaction	0.11
**Cancer recurrence**	
Colorectal	1.15 (0.71-1.87)
Gastric	1.87 (1.28-2.75)
Lung	0.88 (0.49-1.6)
Thyroid	1.6 (0.64-3.98)
Genitourinary tract	2.4 (0.74-7.84)
Others	0.63 (0.35-1.37)
P for interaction	0.42
**All-cause mortality**	
Colorectal	1.12 (0.83-1.5)
Gastric	1.41 (1.04-1.9)
Lung	1.09 (0.69-1.71)
Thyroid	1.04 (0.35-3.05)
Genitourinary tract	2.62 (1.12-6.13)
Others	1.67 (0.94-2.96)
P for interaction	0.21

CI, confidence interval; HR, Hazard ratio; MI, Myocardial infarction; PCI, percutaneous coronary intervention; IPTW, Inverse probability of treatment.

*IPTW included age, sex, income percentile, residential area, clinical presentation at the time of PCI, diabetes, hypertension, dyslipidemia heart failure, cancer type, perioperative cancer treatment and time since cancer diagnosis to surgery.

Table S3. Cardiovascular, bleeding, and cancer outcomes according to the time of prior PCI

	Time from PCI to cancer surgery < 6 months (N = 768)	Time from PCI to cancer surgery ≥6 months (N = 2,853)
**Bleeding requiring transfusion**		
No of cases (IR per 100 py)	474 (18.6)	1304 (14.8)
Crude HR (95% CI)	1.29 (1.16-1.43)	*Reference*
IPTW* HR (95% CI)	1.24 (1.11-1.38)	*Reference*
**Spontaneous MI**		
No of cases (IR per 100 py)	35 (1.4)	75 (0.9)
Crude HR (95% CI)	1.69 (1.13-2.52)	*Reference*
IPTW* HR (95% CI)	2.12 (1.38-3.24)	*Reference*
**Repeat revascularization**		
No of cases (IR per 100 py)	89 (3.5)	212 (2.4)
Crude HR (95% CI)	1.51 (1.18-1.94)	*Reference*
IPTW* HR (95% CI)	1.53 (1.19-1.96)	*Reference*
**Cancer recurrence**		
No of cases (IR per 100 py)	100 (4.1)	279 (3.3)
Crude HR (95% CI)	1.29 (1.02-1.62)	*Reference*
IPTW* HR (95% CI)	1.23 (0.97-1.55)	*Reference*
**All-cause mortality**		
No of cases (IR per 100 py)	207 (8.7)	524 (6.0)
Crude HR (95% CI)	1.39 (1.19-1.64)	*Reference*
IPTW* HR (95% CI)	1.26 (1.06-1.49)	*Reference*

CI, confidence interval; HR, Hazard ratio; MI, Myocardial infarction; PCI, percutaneous coronary intervention; IPTW, Inverse probability of treatment.

*IPTW includes age, sex, income percentile, residential area, clinical presentation at the time of PCI, diabetes, hypertension, heart failure, cancer type, surgical risk, and time from cancer diagnosis to surgery

Table S4. Baseline Characteristics according to surgical timing among patients receiving recent PCI (<12months)

	Time cancer diagnosis to surgery <1 month (Early Surgery) (N = 681)	Time cancer diagnosis to surgery ≥1 month (Late Surgery) (N = 477)	P-value
**Age, years**	67.5 (9.4)	68.2 (8.8)	0.227
**Sex, male**	497 (73.0)	400 (83.9)	<0.001
**Income percentile**			0.282
Medical aid	51 (7.5)	26 (5.5)	
>30th	110 (16.2)	92 (19.3)	
30th – 70th	240 (35.2)	179 (37.5)	
< 70th	265 (38.9)	173 (36.3)	
Unknown	15 (2.2)	7 (1.5)	
**Residential area,** metropolitan	426 (62.6)	295 (61.8)	0.854
**Ever smoker (N = 759)**	107 (56.9)	87 (46.8)	0.240
**Comorbidities**			
Charlson Comorbidities index	6.34 (2.69)	6.99 (2.68)	<0.001
Diabetes	435 (63.9)	316 (66.2)	0.442
Hypertension	157 (23.1)	88 (18.4)	0.069
Dyslipidemia	616 (90.5)	435 (91.2)	0.745
Heart failure	47 (6.9)	33 (6.9)	0.999
**Clinical presentation at the time of PCI, AMI**	178 (26.1)	103 (21.6)	0.088
**Antiplatelet therapy**	617 (90.6)	445 (93.3)	0.127
Aspirin	296 (43.5)	230 (48.2)	0.124
Clopidogrel	306 (44.9)	214 (44.9)	0.999
Prasugrel	4 (0.6)	2 (0.4)	0.999
Ticagrelor	19 (2.8)	14 (2.9)	0.999
**Cancer type**			<0.001
Colorectal	191 (28.0)	92 (19.3)	
Gastric	226 (33.2)	237 (49.7)	
Lung	23 (3.4)	24 (5.0)	
Thyroid	97 (14.2)	47 (9.9)	
Genitourinary tract	118 (17.3)	52 (10.9)	
Others	26 (3.8)	25 (5.2)	
**ESC/ESA Surgical risk**			0.005
Low	131 (8.4)	66 (6.7)	
Moderate-high	575 (84.4)	425 (89.1)	
**Time PCI to surgery, months**			
Median (IQR)	2.5 (1.3-5.6)	4.2 (1.5-8.1)	<0.001
Mean (SD)	5.0 (3.8)	3.8 (3.2)	<0.001
**Time cancer diagnosis to surgery, months**			
Median (IQR)	0.1 (0.0-0.5)	2.1 (1.9-3.2)	<0.001
Mean (SD)	0.3 (0.3)	2.7 (2.0)	<0.001

Values were presented n (%), mean (standard deviation) or median (interquartile range)
AMI, acute myocardial infarction; ESC/ESA, European Society of Cardiology/European Society of Anaesthesiology; PCI, percutaneous coronary intervention.

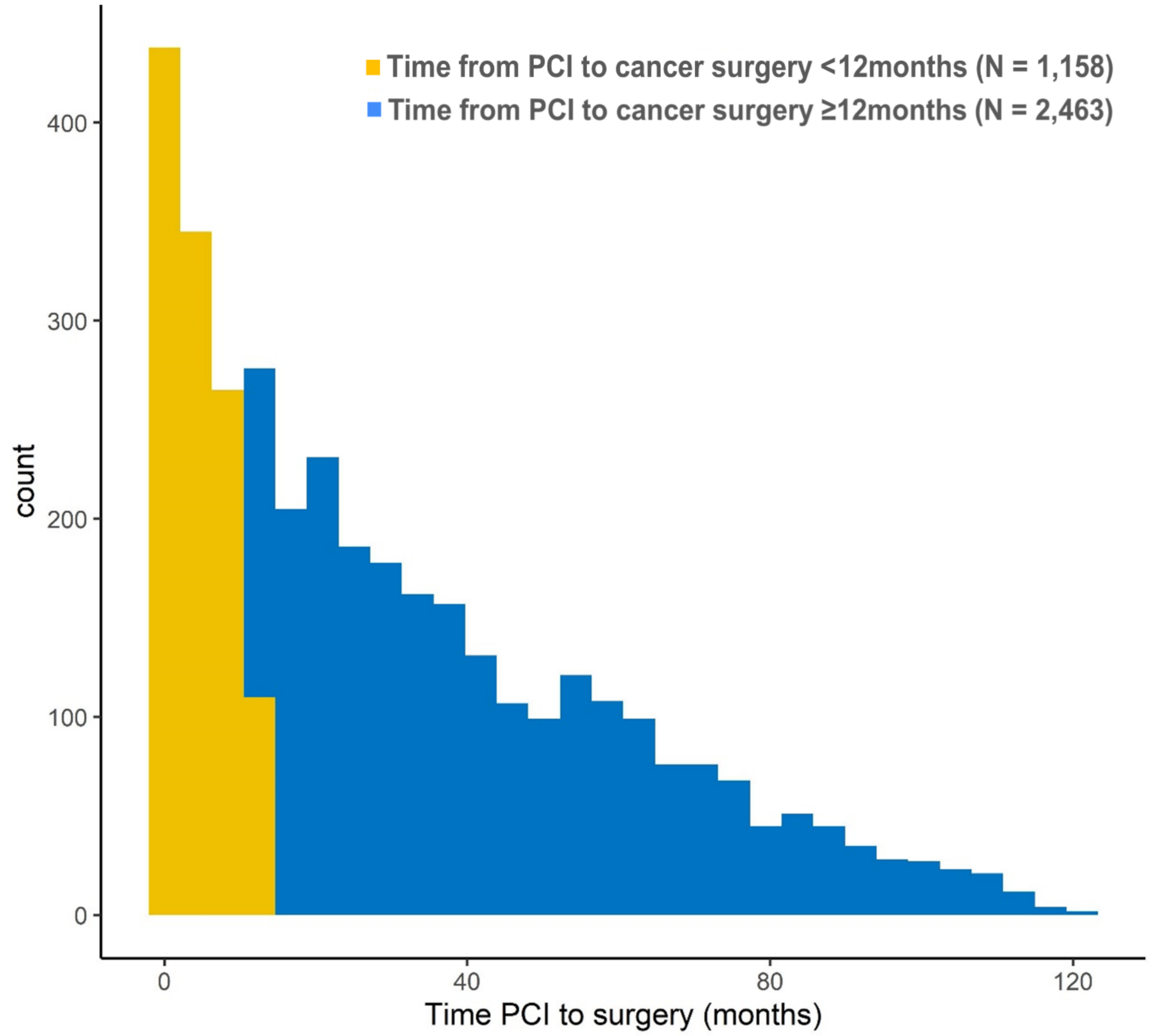

Figure S1. Distributions of the time from PCI to cancer surgery in patients

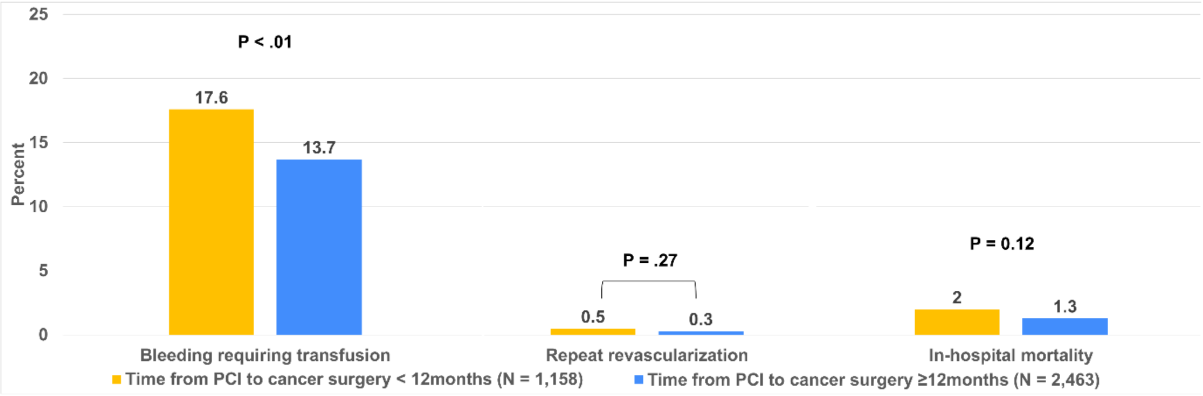

Figure S2. In-hospital outcomes by time from PCI to cancer surgery
